# PICALM rescues glutamatergic neurotransmission, behavioural function and survival in a *Drosophila* model of Aβ42 toxicity

**DOI:** 10.1093/hmg/ddaa125

**Published:** 2020-06-27

**Authors:** Yifan Yu, Teresa Niccoli, Ziyu Ren, Nathaniel S Woodling, Benjamin Aleyakpo, Gyorgy Szabadkai, Linda Partridge

**Affiliations:** 1 Department of Genetics, Evolution and Environment, Institute of Healthy Ageing, University College London, London WC1E 6BT, UK; 2 UK Dementia Research Institute at UCL, London WC1E 6BT, UK; 3 Department of Cell and Developmental Biology, Consortium for Mitochondrial Research, University College London, London WC1E 6BT, UK; 4 The Francis Crick Institute, London NW1 1AT, UK; 5 Department of Biomedical Sciences, University of Padua, Padua 35131, Italy; 6 Max Planck Institute for Biology of Ageing, Cologne 50931, Germany

## Abstract

Alzheimer’s disease (AD) is the most common form of dementia and the most prevalent neurodegenerative disease. Genome-wide association studies have linked *PICALM* to AD risk. PICALM has been implicated in Aβ_42_ production and turnover, but whether it plays a direct role in modulating Aβ_42_ toxicity remains unclear. We found that increased expression of the *Drosophila PICALM* orthologue lap could rescue Aβ_42_ toxicity in an adult-onset model of AD, without affecting Aβ_42_ level. Imbalances in the glutamatergic system, leading to excessive, toxic stimulation, have been associated with AD. We found that Aβ_42_ caused the accumulation of presynaptic vesicular glutamate transporter (VGlut) and increased spontaneous glutamate release. Increased lap expression reversed these phenotypes back to control levels, suggesting that lap may modulate glutamatergic transmission. We also found that lap modulated the localization of amphiphysin (Amph), the homologue of another AD risk factor BIN1, and that Amph itself modulated postsynaptic glutamate receptor (GluRII) localization. We propose a model where PICALM modulates glutamatergic transmission, together with BIN1, to ameliorate synaptic dysfunction and disease progression.

## Graphical Abstract

**Graphical Abstract:**
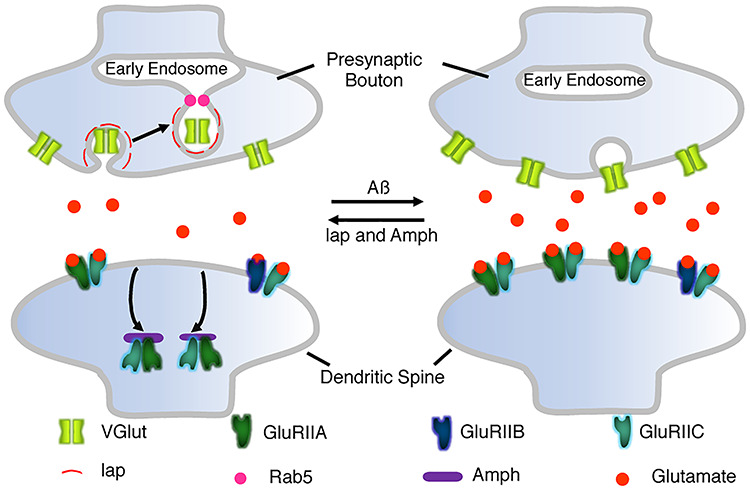
lap and Amph in glutamate metabolism. Aβ affects the localization of both VGlut and GluRIIA, leading to increased glutamate signalling. Lap and Amph restore glutamate signalling, potentially by promoting presynaptic endocytosis of VGlut via lap and/or Rab5/EndoA, and postsynaptic retrival of GluRIIA via Amph.

## Introduction

Alzheimer’s disease (AD) is the most common neurodegenerative disease. Its symptoms include progressive memory loss, cognitive impairment, difficulties in abstract reasoning and decision-making and complete social dependence. AD is characterized by two main neuropathological hallmarks, extracellular amyloid plaques, composed of amyloid-β peptides (Aβ), and intracellular neurofibrillary tangles of hyperphosphorylated tau protein (1).

Most cases of AD are sporadic and strongly age-related. Several genome-wide association studies (GWAS) have linked specific genetic variants to sporadic AD. One of these has been linked to phosphatidylinositol-binding clathrin assembly protein (*PICALM*) and another to bridging integrator 1 (*BIN1*), both of which are involved in clathrin-dependent endocytosis (2,3). PICALM interacts with AP2 and clathrin to recruit cargos to membranes (4). It also physically binds to the soluble NSF attachment protein receptors (SNAREs), such as VAMP2 and VAMP8, and interacts with Rab5 and Rab11 to guide synaptic vesicles from the plasma membrane to endosomes (5–8). PICALM expression is decreased in the brains of AD patients (9,10). However, the role of *PICALM* in AD remains controversial. On the one hand, PICALM knockdown can reduce Aβ_42_ generation, by limiting APP or γ-secretase internalization, and promotes tau degradation, by triggering autophagy (6,11–13). On the other hand, PICALM overexpression has been reported to modulate APP degradation via elevated autophagy or Aβ_42_ clearance through transcytosis (4,8). Although these studies have implicated PICALM in Aβ_42_ production and turnover (11), little is known of its contribution to modulating Aβ toxicity.

BIN1 plays a crucial role in intracellular endosome trafficking, through the interaction with the GTPase dynamin (14). Mice lacking *BIN1* present learning deficits (15). It is not clear whether BIN1 is increased or decreased in AD (16,17), and its role in AD pathology is still unclear. It has been implicated mainly in Tau pathology (18), although its exact role remains to be elucidated. Some studies suggest that the upregulation blocks Tau spread (19), others that downregulation ameliorates Tau toxicity (17). BIN1 also plays a role in Aβ production, with reduced expression linked to increased BACE1 and Aβ production (20,21). However, whether BIN1 plays a role in Aβ toxicity has also not been investigated. Recently, BIN1 was shown to be involved in neurotransmitter release in mouse hippocampal neurons (22).

Glutamate excitotoxicity has long been thought to play an important role in AD aetiology (23). Glutamatergic neurons are severely affected in AD, and it has been speculated that the disease might be caused, at least in part, by over-activation of glutamatergic neurons (24). Aβ oligomers enhance glutamate release (25–27) and impair glutamate reuptake by astrocytes (28,29), leading to increased extracellular glutamate and activation of extra-synaptic NMDAR receptors and synaptic damage (25,30). Aβ also affects the composition of glutamate receptors (GluRII), with a reduction of GluA1 and GluA2 subunits of the AMPA receptor, and both GluN1 and GluN2A of the NMDA receptor (31–34). Up-regulation of these has been linked to suppression of Aβ toxicity (35–38). On the other hand, downregulation of GluA3 or GluN2B can ameliorate Aβ toxicity (25,30,39). These studies suggest that amyloid beta peptides (Aß) can exert neurotoxic effects both through increased glutamatergic excitotoxicity and through altered composition of postsynaptic glutamate receptors.

Here we demonstrate that overexpression of the *Drosophila* PICALM orthologue, like AP180 (lap), ameliorates Aβ42-induced shortened lifespan and locomotor defects in a fly AD model, importantly without affecting Aβ42 levels. Because these findings implicated a gene involved in endocytosis/exocytosis in Aβ42 toxicity, we next performed a small-scale, targeted, genetic screen of endocytic/exocytic genes (Table 1) and identified Rab5 and EndoA as partial suppressors of Aβ42-induced toxicity. Given that Rab5 and EndoA are involved in cargo translocation from the plasma membrane to the early endosome, our findings suggested that early steps in endocytosis, including those mediated by lap, are crucial to AD progression. Aβ expression also led to the accumulation of vesicular glutamate transporters (VGlut) at the presynaptic region, and increased lap expression restored their wild-type localization. Concordantly, lap reduced the increased glutamate release during spontaneous activity associated with Aβ expression. lap also restored the localization of the BIN1 orthologue, amphiphysin (Amph), which was disrupted upon Aβ expression. Moreover, Amph modulated the localization of glutamate receptors (GluRII), which was also disrupted by Aβ expression. We therefore propose a novel model where PICALM and BIN1 can cooperate to restore the distribution of pre- and postsynaptic proteins involved in glutamatergic neurotransmission and thus ameliorate aberrant glutamatergic transmission and neurotoxicity in the presence of Aβ42.

**Table 1:** Screen for endocytic–exocytic genes on *Drosophila* lifespan

Drosophila homologue	Human gene	Function	Effect on lifespan
Cindr	CD2AP	Actin remodelling	Decreased
Clc	CLC	Vesicle formation	Decreased
Shi	DYNAMIN	Vesicle scission	None
EndoA	ENDOA	Vesicle formation	Increased
Eps15	EPS15	Vesicle formation	Decreased
Iqf	EPSIN	Vesicle formation	Decreased
Rab4	RAB4	Endocytic recycling	Decreased
Rab5	RAB5	Plasma membrane to early endosome	Increased
Rab7	RAB7	Early-to-late endosome	Decreased
Rab8	RAB8	Endocytic recycling	Decreased
Rab10	RAB10	Endocytic recycling	Decreased
Rab11	RAB11	Endocytic recycling	Decreased
Rab14	RAB14	Endocytosis	None
Snap25	SNAP25	Vesicle fusion	None
nSyb	VAMP2	Vesicle fusion	None

Summary table of the effect of overexpression of a number of genes on the lifespan of Aß-expressing flies.

## Results

### lap reduces Aβ42 pathology

**Figure 1:**
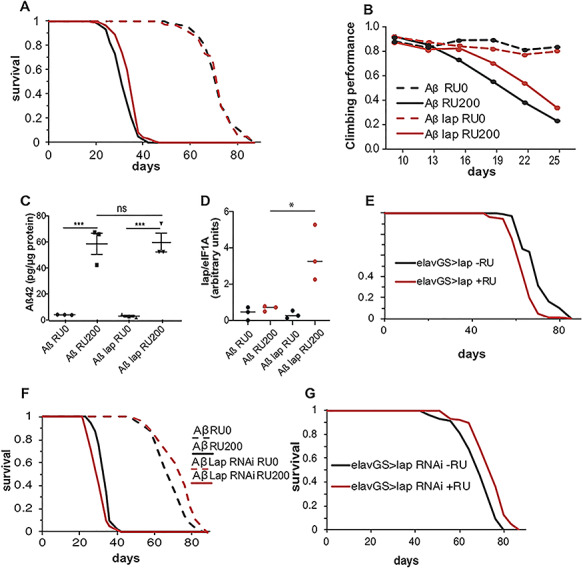
lap alleviates Aβ_42_ toxicity. (**A**) Survival curves of flies expressing Aβ, with (red lines, UAS-Aβ/UAS-lap; elavGS/+) and without (black lines, UAS-Aβ/+; elavGS/+) lap co-overexpression, in adult neurons (RU200, solid lines) and uninduced controls (RU0, dashed lines). Expression of Aβ in neurons shortened lifespan, and lap co-overexpression significantly improved this shortened lifespan. *n* > 120 per condition. Aβ RU0 versus Aβ RU200, *P* = 1.1E−72; Aβ RU0 versus Aβ lap RU0, ns, not significant; Aβ lap RU0 versus Aβ lap RU200, *P* = 2.44E−47; Aβ RU200 versus Aβ lap RU200, *P* = 1.8E−5, determined by log–rank test. There was a significant interaction of genotype and RU by Cox proportional hazard analysis, *P* = 0.016, indicating that expression of lap significantly extended the lifespan of Aß-expressing flies. (**B**) Locomotor performance index of flies of the same genotypes as in (A). Aβ caused a climbing defect, which was significantly improved by the co-overexpression of lap, *n* = ~50 flies per condition. There was a statistically significant interaction between RU and genotype by ordinal logistics, *P* = 0.00040826, indicating that the expression of lap significantly improved the climbing of Aß-expressing flies. (**C**) Aβ_42_ protein levels, measured by ELISA, in the heads of 21-day-old flies expressing Aβ with or without co-overexpression of lap in neurons (RU200) and uninduced controls (RU0). lap co-overexpression had no effect on Aβ_42_ levels. Means ± SEM, *n* = 3 biological replicates of 10 heads per replicate per condition. *F*(3,8) = 34.53, *P* < 0.0001 by one-way ANOVA;^*^^*^^*^P < 0.01, ns, not significant, comparison by Tukey’s *post hoc* test. (**D**) qPCR of lap mRNA levels in fly heads expressing Aß, Aß and lap (RU200) and their uninduced controls (RU0), showing overexpression of lap in the Aß, lap expressing flies (*F*(3,8) = 1.987) by one-way ANOVA ^*^P = 0.0088 for comparison between the two induced conditions. (**E**) Adult survival curves of lap overexpression in adult neurons (RU200, red line) and uninduced controls (RU0, black line), *n* > 120 per condition, *P* = 4.5E−16, by log–rank test. (**F**) Adult survival of flies harbouring Aβ with (red lines, UAS-Aβ/+; elavGS/UAS-lap-RNAi) or without (black lines, UAS-Aβ/+; elavGS/+) lap RNAi in adult neurons (RU200, solid lines) and uninduced controls (RU0, dashed lines). Inhibition of lap reduces longevity of Aβ-expressing flies. *n* > 120 per condition. Aβ RU0 versus Aβ RU200, *P* = 7.6254E−72; Aβ RU0 versus Aβ lap RU0, *P* = 1.48871E−06; Aβ lap RU0 versus Aβ lap RU200, *P* = 1.65991E−69; Aβ RU200 versus Aβ lap RU200, *P* = 3.2225E−10, by log–rank test. There was a significant interaction of genotype and RU by Cox proportional hazard analysis, *P* = 9.5E−06, indicating that downregulation of lap significantly shortened the lifespan of Aß-expressing flies. (**G**) Adult survival curves of lap RNAi flies in adult neurons (RU200, red line) and uninduced controls (RU0, black line), *n* > 120 per condition. *P* = 1.08638E−09, by log–rank test.

To explore the role of *PICALM* in AD aetiology *in vivo*, we examined the role of the *Drosophila* homologue *lap* (40) in Aβ pathology*.* We generated an adult-onset model of Aβ toxicity that expressed two copies of wild-type Aβ_42_ (41) under the control of an inducible, pan-neuronal driver (*elavG*S) (42). ElavGS is induced by the drug RU486 (42), which was added to the fly food only after eclosion, thus restricting the expression of Aβ to adult neurons. The Aβ_42_-expressing flies had a shortened lifespan and displayed locomotor deficits (Fig. 1A and B), suggesting that wild-type Aβ_42_ is toxic to adult neurons, as previously reported (43). In humans, two SNPs near the gene PICALM, rs3851179 and rs541458, which are associated with decreased levels of AD occurrence in a number of patient cohorts (44,45) are associated with higher levels of expression of PICALM (using the LIBD eQTL browser (46)), potentially suggesting that PICALM has a protective role in AD. To test this, we generated flies overexpressing *lap* under the control of the UAS promoter and confirmed the overexpression under induction of ElavGS by qPCR (Fig. 1D). Co-overexpression of *lap* in Aβ_42_-expressing flies attenuated Aβ_42_ toxicity, ameliorating both the reduction in lifespan and the impaired locomotion as assessed by negative geotaxis (climbing) assays (Fig. 1A and B). Importantly, increased lap expression did not alter Aβ_42_ protein levels (Fig. 1C), suggesting that lap acts downstream of Aβ_42_ generation or degradation. Conversely, inhibition of *lap* by RNA interference enhanced Aβ_42_ toxicity, leading to further shortening of lifespan (Fig. 1F).

The protective allele for SNP rs3851179 is also enriched in Italian centenarians, suggesting a role for PICALM/lap in healthy ageing as well as AD (47). We therefore examined the effect of *lap* on healthy ageing, by both overexpressing and downregulating its expression in neurons in the absence of Aβ_42_. In contrast to our findings with Aβ42 expression, *lap* overexpression on its own shortened lifespan, while RNAi against *lap* extended lifespan (Fig. 1E and G), indicating that the neuroprotective effect of *lap* overexpression was specific to Aβ toxicity and not due to a broader effect on ageing.

### lap regulates glutamate release

**Figure 2:**
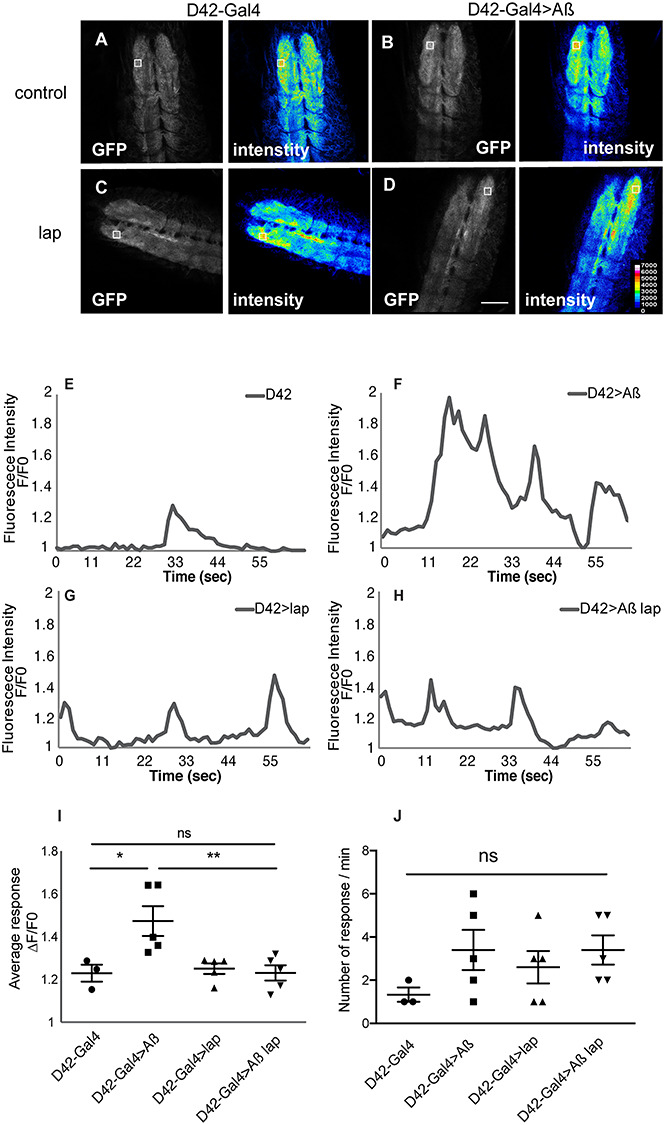
Lap reduces excess glutamate release upon Aβ expression. (**A**–**D**) Single confocal images from movies of intact L2 larvae expressing iGluSnFR in neurons, showing local and transient increases in fluorescence reflecting the release of glutamate. The image is a representative burst of fluorescence from a movie (in grey) and an intensity map of the same frame (in colour). Genotypes: (A) D42-Gal4/UAS-iGluSnFR, (B) UAS-Aβ/+; D42-Gal4/UAS-iGluSnFR, (C) UAS-lap/+; D42-Gal4/UAS-iGluSnFR, (D) UAS-Aβ/UAS-lap; D42-Gal4/UAS-iGluSnFR. Scale bar, 20 μm. (**E**–**H**) Traces of changes in iGluSnFR fluorescence display the spontaneous activity in the neuropil. Fluorescence signals are normalized to minimum fluorescence in each trace and expressed relative to baseline. Genotypes are as above. The arrow indicates the time point shown in (A–D) (**I**) Quantification of the amplitude of the glutamate burst (described as changes in fluorescence relative to baseline) in the neuropil of L2 larvae shown in A–D, expressing driver alone with the reporter or together with Aβ, lap or both driven, plotted as means per animal ± SEM, *n* > 3 animals. Genotypes are as above. *F*(3,14) = 6.503, *P* = 0.0056, determined by one-way ANOVA; ^*^^*^P < 0.001, ns, not significant, comparison by Tukey’s *post hoc* test. (**J**) Quantification of the number of glutamate release events of the same larvae in (I), plotted as means ± SEM, *n* > 3 per condition. Genotypes: D42-Gal4/UAS-iGluSnFR, UAS-Aβ; D42-Gal4/UAS-iGluSnFR, UAS-lap; D42-Gal4/UAS-iGluSnFR, UAS-Aβ/UAS-lap; D42-Gal4/UAS-iGluSnFR. *F*(3,14) = 1.248, *P* = 0.3299, determined by one-way ANOVA; ns, not significant, comparison by Tukey’s *post hoc* test.

AD is associated with glutamate excitotoxicity (23,48), and Aβ oligomers lead to increased glutamate release (25). To check if this was also the case in our fly model, we used the fluorescent extracellular glutamate reporter intensity-based glutamate sensing fluorescent reporter (iGluSnFR) to detect extracellular glutamate levels (49,50). We expressed UAS-iGluSnFR in larval motor neurons and observed transient, local bursts of fluorescence, presumably associated with spontaneous local release of glutamate (Fig. 2A and E). We occasionally also observed waves of fluorescence running along the anterior–posterior axis, similar to those previously reported (50) (Supplementary Material, Movie S1). Expression of Aβ led to a dramatic increase in the intensity of local bursts (Supplementary Material, Movie S2, Fig. 2B and F), suggesting that Aβ_42_ increased the release of glutamate, consistent with a previous study observing increased glutamate neurotransmission in APP/PS1 transgenic mice (51). However, we did not observe any change in the number of glutamate release events per minute with Aβ expression (Fig. 2J). Strikingly, lap co-expression reduced the intensity of local fluorescence bursts back to control levels (Fig. 2D, H, I and Supplementary Material, Movie S4), whereas expression of lap alone has no effect (Fig 2C, G, I and Supplementary Material, Movie S3). Taken together, these findings suggest that Aβ compromises components of glutamatergic signalling, leading to increased glutamate release, and that lap overexpression acts to reinstate healthy levels of glutamatergic signalling.

**Figure 3:**
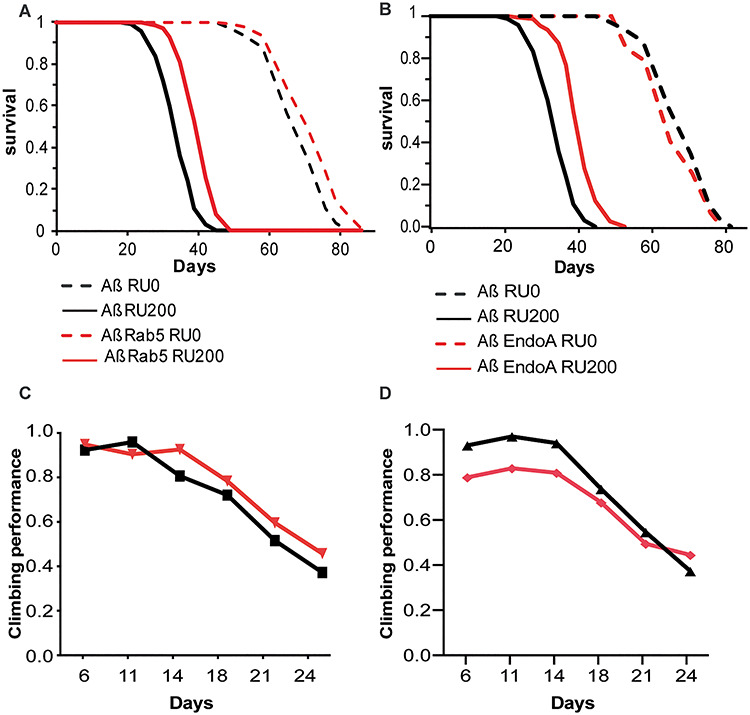
Rab5 and Endo A ameliorates Aβ_42_ toxicity. (**A**) Survival curves of flies expressing Aβ, with (red lines, UAS-Aβ/+; elavGS/UAS-Rab5) and without (black lines, UAS-Aβ/+; elavGS/+) Rab5 co-overexpression, in adult neurons (RU200, solid lines) and uninduced controls (RU0, dashed lines). Co-overexpression of Rab5 extended lifespan of Aβ-expressing flies. *n* > 120 per condition. Aβ RU0 versus Aβ RU200, *P* = 7.46192E−76; Aβ RU0 versus Aβ Rab5 RU0, *P* = 6.07338E−05; Aβ Rab5 RU0 versus Aβ Rab5 RU200, *P* = 1.54498E−70; Aβ RU200 versus Aβ Rab5 RU200 *P* = 6.06826E−22, determined by log–rank test. There was a significant interaction of genotype and RU by Cox proportional hazard analysis, *P* = 0.000338, indicating that expression of Rab5 significantly extended the lifespan of Aß-expressing flies. (**B**) Survival curves of flies expressing Aβ, with (red lines, UAS-Aβ/UAS-EndoA; elavGS/+) and without (black lines, UAS-Aβ/+; elavGS/+) EndoA co-overexpression, in adult neurons (RU200, solid lines) and uninduced controls (RU0, dashed lines). Co-overexpression of EndoA extended lifespan of Aβ-expressing flies. *n* > 120 per condition. Aβ RU0 versus Aβ RU200, *P* = 7.46192E−76; Aβ RU0 versus Aβ EndoA RU0, *P* = 0.007893292; Aβ EndoA RU0 versus Aβ EndoA RU200, *P* = 5.2333E−70; Aβ RU200 versus Aβ EndoA RU200 *P* = 1.42894E−26, determined by log–rank test. There was a significant interaction of genotype and RU by Cox proportional hazard analysis, *P* < 2E−16, indicating that expression of EndoA significantly extended the lifespan of Aß-expressing flies. (**C**) Locomotor performance index of induced flies of the same genotypes as in (A). Co-overexpression of Rab5 did not effect climbing ability by ordinal logistics regression (*P* = 0.62), *n* = ~50 flies per condition. (**D**) Locomotor performance index of induced flies of the same genotypes as in (B). Co-overexpression of EndoA worsened the climbing ability of Aß flies but slowed down the decline relative to Aß-expressing flies alone, *P* = 3.3867E−06 for effect of genotype (*z* value = −4.6), *P* = 0.00011043 for interaction between genotype and time (*z* value = 3.9), *n* = ~50 flies per condition.

Next we investigated the molecular mechanisms by which *lap* reduced Aβ_42_ toxicity. *PICALM* plays a major role in endocytosis (52,53), which is important for accurate neurotransmitter signalling. To determine whether endocytosis could play a wider role in the rescue of Aβ toxicity, we performed a targeted genetic screen of well-characterized components regulating endocytosis (Table 1). Of the genes tested, only the overexpression of Rab5 and EndoA ameliorated the shortened lifespan (Fig. 3A and B). However, Rab5 did not ameliorate climbing (Fig. 3C), while EndoA worsened the overall climbing ability of Aß-expressing flies but slowed down the rate of decline, suggesting it was slowing the development of Aß toxicity but possibly had some direct detrimental effect on climbing. In contrast, the overexpression of *Rab4*, *Rab7*, *Rab8*, *Rab10* and *Rab11* exacerbated Aβ42 toxicity (Table 1). These findings suggest that the upregulation of early steps of clathrin-mediated endocytosis up to the early endosome could play some part in the amelioration of Aβ toxicity, consistent with a study in yeast (54). However, given the contrasting effects we observed for different Rab genes in our small screen, we hypothesized that lap’s rescue could be mediated by additional key factors.

### lap mediates VGlut localization

**Figure 4:**
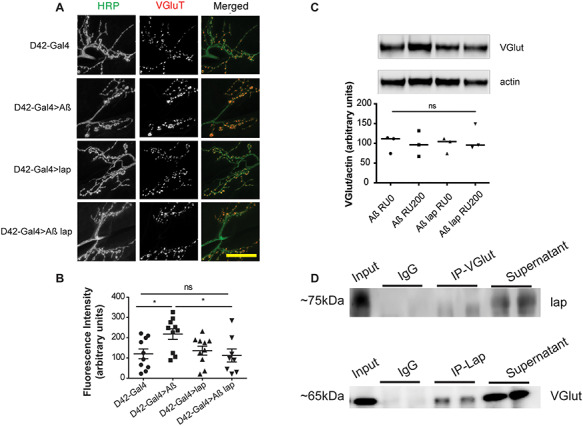
Lap reduces the Aβ-induced accumulation of VGlut at the larval NMJ. (**A**) Confocal images of the NMJs of wandering third-instar larvae expressing the D42-Gal4 driver alone (+/+; D42-Gal4/+), and Aβ (UAS-Aβ/+; D42-Gal4/+), lap (UAS-lap/+; D42-Gal4/+), or Aβ + lap (UAS-Aβ/UAS-lap; D42-Gal4/+) driven by D42-Gal4. Scale bar, 20 μm. (**B**) Fluorescence intensity scores are plotted as means ± SEM, *n* > 7 per condition. *F*(3,34) = 3.576, *P* = 0.0238 determined by one-way ANOVA; ^*^P < 0.05, ns, not significant, comparison by Tukey’s *post hoc* test. (**C**) Western blot and quantification of VGlut relative to actin in adult *Drosophila* heads expressing Aß or Aß and lap (RU200) and their uninduced controls (RU 0). Genotypes: UAS-Aβ; elavGS/+, UAS-Aβ/UAS-lap; elavGS/+. (**D**) Western blots of fractions of a co-IP for VGlut (upper) and lap (lower) from the heads of wild-type adult flies, probed for lap (upper) and VGlut (lower). Samples are head extracts before the IP was started (input), bead-only pull-down (IgG) showing no non-specific binding; pull-down with the indicated antibody (IP-VGlut, IP-lap), showing successful pull-down of the binding partner; supernatant after the pull-down (supernatant), showing only partial depletion; please see Materials and Methods for details. These IP show that VGlut can pull-down lap and lap can pull-down VGlut. All co-IPs were run in duplicate.

Defects in endocytosis caused by Aβ have been reported to disrupt the trafficking of transmembrane proteins to their proper destination (54), and alterations in endocytic trafficking can disrupt the delivery or recycling of synaptic proteins (55). lap collaborates with clathrin to recycle synaptic vesicles, regulating the efficiency of synaptic vesicle endocytosis and vesicle size (56). lap is also required for recruitment of synaptic vesicle proteins (40,57) and is known to bind to vesicular glutamate transporters (VGlut) (57). We confirmed this by co-immunoprecipitating VGlut with lap in heads of wild type adult flies (Fig. 4D). VGlut is involved in loading glutamate into synaptic vesicles and regulates glutamate release events. We therefore determined whether the protective role of lap was mediated by VGlut. VGlut expression was not altered by *Aβ_42_* or *lap* expression (Fig. 4C). Next we assessed VGlut distribution. Adult *Drosophila* fly brains have a high density of neurons, making it difficult to monitor individual synapses. We therefore turned to the larval neuromuscular junctions (NMJ), which provide an excellent model system for monitoring individual synapses, and are extensively used to analyse cellular and molecular mechanisms of synaptic development and neurotransmission (58). We observed that VGlut was abnormally accumulated at the presynaptic terminal of the NMJ upon Aβ_42_ expression (Fig. 4A and B), potentially impairing glutamatergic synaptic transmission. This accumulation was reduced by *lap co-*overexpression (Fig. 4A and B), suggesting that lap overexpression acts to reinstate wild-type glutamatergic signalling by directly binding (Fig. 4D) and regulating the localization of vesicular transporters (Fig. 4) and therefore affecting the release of glutamate as previously observed (Fig. 2).

### Postsynaptic loss of Amph is rescued by lap

**Figure 5:**
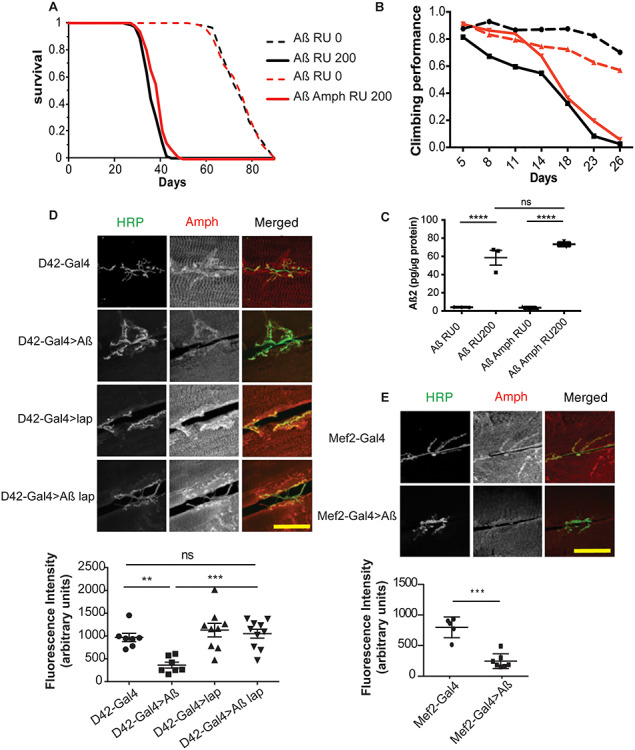
Lap regulates Amph localization. (**A**) Survival curves of flies expressing Aβ, with (red lines, UAS-Aβ/UAS-Amph; elavGS/+) and without (black lines, UAS-Aβ/+; elavGS/+) lap co-overexpression, in adult neurons (RU200, solid lines) and uninduced controls (RU0, dashed lines). Expression of Aβ in neurons shortened lifespan, and lap co-overexpression significantly rescued this shortened lifespan. *n* > 145 per condition. Aβ RU0 versus Aβ RU200, *P* = 3.29E−73; Aβ RU0 versus Aβ Amph RU0, ns, not significant; Aβ Amph RU0 versus Aβ Amph RU200, *P* = 1.25E−82; Aβ RU200 versus Aβ lap RU200, *P* = 8.8E−7, determined by log–rank test. (**B**) Locomotor performance index of flies of the same genotypes as in (A). Aβ caused a climbing defect, which was significantly rescued by co-overexpression of Amph, *n* = ~50 flies per condition. There was a statistically significant interaction between RU and genotype by ordinal logistics, *P* = 1.0908E−12, indicating that expression of Amph significantly improved the climbing specifically of of Aß-expressing flies. (**C**) Aβ_42_ protein levels, measured by ELISA, in the heads of 21-day-old flies expressing Aβ with or without co-overexpression of Amph in neurons (RU200) and uninduced controls (RU0). Amph co-overexpression had no effect on Aβ_42_ levels. Means ± SEM, *n* = 3 biological replicates of 10 heads per replicate per condition. *F*(3,8) = 0.9406, *P* < 0.0001 by one-way ANOVA; ^*^^*^^*^^*^P < 0.001, ns, not significant, comparison by Tukey’s *post hoc* test. (**D**) Confocal images and quantification of Amph fluorescence at the NMJ of wandering third-instar larvae expressing the D42-Gal4 driver alone (+/+; D42-Gal4/+), and Aβ (UAS-Aβ/+; D42-Gal4/+), lap (UAS-lap/+; D42-Gal4/+), or Aβ + lap (UAS-Aβ/UAS-lap; D42-Gal4/+) driven by D42-Gal4, plotted as means ± SEM, *n* > 7 per condition. Scale bar, 20 μm. *F*(3,29) = 8.954, *P* = 0.0002, determined by one-way ANOVA; ^*^^*^P < 0.01, ^*^^*^^*^P < 0.001, ns, not significant, comparison by Tukey’s *post hoc* test. (**E**) Confocal images and quantification of Amph fluorescence at the NMJ of wandering third-instar larvae expressing the Mef2-Gal4 driver alone (Mef2-Gal4/+) or Aβ driven by Mef2-Gal4 (UAS-Aβ/+; Mef2-Gal4/+), plotted as means ± SEM, *n* > 5 per condition. Scale bar, 20 μm. ^*^^*^^*^P < 0.001, comparison by Student’s test.

*BIN1*, another AD modifier identified by GWAS in humans (59), also plays a role in endocytosis (60) and has recently been shown to regulate neurotransmitter release in mouse glutamatergic neurons (22). The overexpression of Amph, the fly homologue of BIN1, led to a slight rescue in lifespan (Fig. 5A) and climbing (Fig. 5B), without affecting Aß levels (Fig. 5C).

In flies, lap is expressed presynaptically (40,56), whereas Amph is expressed postsynaptically (61,62), implying no direct interaction between lap and Amph. To investigate the interplay between them, we assessed Amph localization at the larval NMJ. We expressed Aβ either presynaptically, with D42-Gal4, or postsynaptically, with Mef2-Gal4, and saw that, in both cases, Amph abundance was significantly decreased upon Aβ_42_ expression (Fig. 5D and E). Overexpression of Aß at the NMJ does not affect the bouton size or dendritic branching (63). We therefore hypothesized that the changes in signalling were responsible for changed in Amph localization via a yet unknown mechanism. Overexpression of lap presynaptically rescued Amph localization at the NMJ (Fig. 5D), suggesting that presynaptic lap can affect the postsynaptic localization of Amph, potentially through the effects on VGlut and glutamate release observed above.

### Amph and lap modulate GluR accumulation

**Figure 6:**
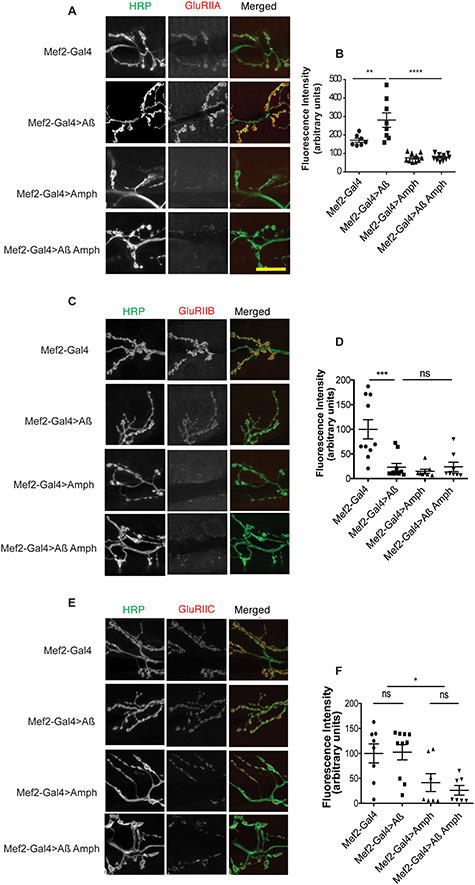
Amph effects GluRII localization. (**A**) Confocal images of the NMJ of wandering third-instar larvae expressing the Mef2-Gal4 driver alone (+/+; Mef2-Gal4/+), and Aβ (UAS-Aβ/+; Mef2-Gal4/+), Amph (Mef2-Gal4/UAS-Amph), or Aβ + lap (UAS-Aβ/+; Mef2-Gal4/UAS-Amph) driven by Mef2-Gal4, stained for GluRIIA. (**B**) Fluorescence intensity scores from (A) are plotted as means ± SEM, *n* > 7 per condition. *F*(3,31) = 25.04, *P* < 0.0001, determined by one-way ANOVA; ^*^^*^P < 0.01, ^*^^*^^*^P < 0.001, ^*^^*^^*^^*^P < 0.0001, comparison by Tukey’s *post hoc* test. (**C**) GluRIIB fluorescence at the NMJ of wandering third-instar larvae expressing driver alone and Aβ, Amph or both driven by Mef2-Gal4 (genotypes as in A). (**D**) Fluorescence intensity scores from (C) are plotted as means ± SEM, *n* > 7 per condition. Scale bar, 20 μm. *F*(3,32) = 10.66, *P* < 0.001, by a one-way ANOVA; ^*^^*^^*^P < 0.001, ns, not significant, by Tukey’s *post hoc* test. (**E**) GluRIIC fluorescence at the NMJ of wandering third-instar larvae expressing driver alone, and Aβ, Amph or both driven by Mef2-Gal4 (genotypes as in A). (**F**) Fluorescence intensity scores from (E) are plotted as means ± SEM, *n* > 7 per condition. Scale bar, 20 μm. *F*(3,28) = 5.847, *P* = 0.0031 by one-way ANOVA; ^*^P < 0.05, ns, not significant, by Tukey’s *post hoc* test.

Amph modulates the localization of Dlg, the *Drosophila* homologue of PSD-95, which is known to stabilize glutamate receptors (61,62). The fly NMJ ionotropic glutamate receptor GluRII is composed of regulatory subunits GluRIIA, GluRIIB and constitutive GluRIIC, with GluRIIA increasing postsynaptic sensitivity and GluRIIB decreasing it (64–67). We determined whether Aβ_42_ regulated GluRII localization in larvae by expressing Aβ_42_ postsynaptically, and we found that GluRIIA substantially accumulated on the plasma membrane while GluRIIB decreased (Fig. 6A–D), in line with previous studies showing that the upregulation of GluRIIA leads to a decrease in GluRIIB (68). GluRIIC localization remained unchanged (Fig. 6E). These findings suggest that Aβ expression can alter the composition of GluRII receptors postsynaptically.

We next assessed whether increased Amph expression could rescue these deficits in the composition of GluRII receptors. However, we found that overexpression of Amph led to a dramatic decrease in the localization of all the GluRII subunits at the NMJ (Fig. 6), indicating that Amph likely modulates glutamate receptor localization rather than composition.

Lap and Amph therefore both regulate the localization of key components of glutamatergic signalling, which could contribute to their rescue of Aβ toxicity.

**Figure 7:**
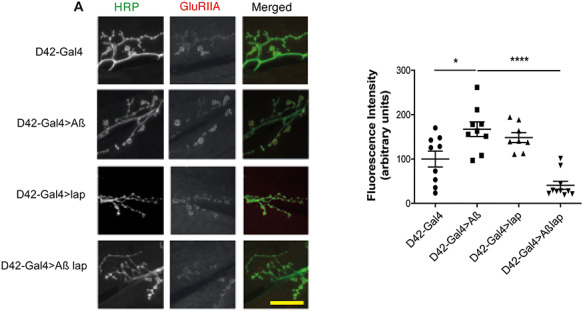
Lap affects GluRII localization. (**A**) Confocal images and (**B**) quantification of GluRIIA fluorescence at the NMJ of wandering third-instar larvae expressing the D42-Gal4 driver alone (+/+; D42-Gal4/+), and Aβ (UAS-Aβ/+; D42-Gal4/+), lap (UAS-lap/+; D42-Gal4/+), or Aβ + lap (UAS-Aβ/UAS-lap; D42-Gal4/+) driven by D42-Gal4, plotted as means ± SEM, *n* > 8 per condition. Scale bar, 20 μm. *F*(3,32) = 1.558, *P* ≤ 0.0001, determined by one-way ANOVA; ^*^P < 0.05, ^*^^*^^*^^*^P < 0.0001, ns, not significant, comparison by Tukey’s *post hoc* test.

Given Amph expression postsynaptically affects the localization of GluRIIA while Aβ_42_ and lap expression presynaptically affects Amph localization postsynaptically, we checked whether the expression of Aß and lap presynaptically affected GluRIIA. Indeed the expression of Aβ_42_ presynaptically increased GluRIIA levels postsynaptically, and co-overepxression of lap reduced GluRIIA levels (Fig. 7), suggesting that lap, via Amph, can affect GluRIIA localization. How this is mediated will require further investigation.

## Discussion

Several publications have described the role of *PICALM* in Aβ_42_ production and clearance. However, its role in Aβ_42_ toxicity remains less explored. Our findings provide a novel link between the *Drosophila* homologues of *PICALM* and *BIN1*, *lap* and *Amph*, respectively, and glutamatergic transmission in an AD model.

We showed that Aβ expression leads to an increase in spontaneous local burst of glutamate, possibly because of an increase in presynaptic VGlut, which could lead to excessive glutamate release (69). Based on the known effects of VGlut overexpression in increasing vesicle size rather than altering glutamate concentration within the vesicle (69), and the ability of lap to reduce vesicle size (56), it is likely that increased presynaptic vesicle size is one mechanism by which Aβ increases glutamate release. In addition, we find that Aβ expression increases postsynaptic GluRIIA, which can lead to an alteration in postsynaptic sensitivity of the GluRII receptor (67). Both of these changes could lead to aberrant glutamatergic signalling and neurotoxicity. Defects in glutamatergic signalling are a key feature of AD (23), with expression of glutamate receptors and transporters altered in sporadic AD patients (28) and mouse AD models (70). Furthermore, treatment of mouse hippocampal slices and cultured neurons with Aß oligomers leads to excess extracellular glutamate (71,72). We have shown that lap and Amph interact from both sides of the synapse to restore wild-type levels and localization of effectors of glutamatergic synaptic transmission. lap decreases VGlut levels presynaptically, whereas Amph lowers GluRIIA levels postsynaptically. Moreover, lap can restore postsynaptic Amph localization, which is disrupted by Aβ expression, suggesting that lap, via Amph, could possibly modulate GluRIIA localization too.

lap has been shown to bind VGlut and mediate its endocytosis from the plasma membrane (57). We also showed that Rab5 and EndoA, which play a role in mediating the formation of clathrin coated pits at the plasma membrane (73), as well as in fusion of endocytic vesicles with early endosomes (74,75), could also rescue Aβ_42_ shortened lifespan. This finding suggests that lap’s endocytic function may contribute to its ability to rescue Aβ_42_ toxicity, by removing excess VGlut from the synaptic terminal. In contrast, PICALM endocytic function has not been directly linked to synaptic vesicle proteins or to glutamatergic signalling, and it will be interesting to determine whether PICALM plays a similar role in mammalian AD models and can directly modulate Aβ_42_ toxicity. It is interesting to note the opposing effect of lap in normal ageing as opposed to a disease context. It is possible that, since glutamate receptors have been shown to decrease during ageing (76,77), a decrease in lap and possibly also endocytosis helps maintain glutamate signalling in the context of ageing, but in a pathological context characterized by excess glutamate signalling, an increase in lap is beneficial. One caveat in our experiments is we overexpressed a single lap isoform, whereas in flies there are nine isoforms; it will be interesting to determine the effect of overexpressing the other isoforms.

Amph also plays an important role in endocytosis, but its role in Aβ toxicity remains unexplored. Our study highlights a role for Amph in regulating the localization of glutamate receptor GluRII at the synapse. GRIK4, the human homologue of GluRIIA, is increased in AD patients (78), and it would therefore be interesting to verify whether BIN1 can modulate its localization.

In summary, we identified a novel role of two prominent AD-associated GWAS hits, PICALM and BIN1, as modulators of glutamatergic signalling, which could contribute to their role in AD aetiology. It would be interesting to investigate whether this role is conserved in mammalian models of AD, thus potentially opening the possibility of targeting PICALM and BIN1 as modulators of Aβ toxicity in sporadic AD.

## Materials and Methods

### Fly husbandry and stocks

All flies were reared at 25°C on a 12-h:12-h light–dark (LD) cycle at constant humidity and on standard sugar-yeast-agar (SYA) medium (agar, 15 g/l; sugar, 50 g/l; autolyzed yeast, 100 g/l; nipagin, 30 ml/l (of 10% solution in ethanol) and propionic acid, 2 ml/l). ElavGS, derived from the original elavGS 301.2 line (42), was a gift from Dr H. Tricoire (CNRS), the UAS-Aβ42 stock was a gift from Dr P. Fernandez-Funez (University of Minnesota, Twin Cities, USA) (41), and the UAS-Cindr line was a gift from Dr H. Stenmark (University of Oslo, Oslo, Norway) (79). All UAS lines for the genetic screen (including UAS-Rab5-GFP and UAS-Amph), lap RNAi (TRiP.HMS01939), D42-Gal4, Mef2-Gal4 and iGluSnFR were obtained from the Bloomington *Drosophila* Stock Center. UAS-Rab8-HA and UAS-Rab11-HA were obtained from the FlyORF. The Amph RNAi (v9264) line was obtained from the Vienna *Drosophila* RNAi Center.

To generate the UAS-lap transgenic line, a 1.4-kb DNA fragment containing the lap A isoform was amplified by PCR using primers CACCATGACCATGGCAGGG and TTACTGTGCGGCGCCG from cDNA clone RH47395 from Drosophila Genomics Resource Center (NIH Grant 2P40OD010949), gateway cloned into an entry vector and transferred into a gateway compatible pUAST vector according to standard protocols (80). The UAS-lap construct was randomly inserted into the *w^1118^* background, and two clones on chromosome 2 were used for the analysis. To ensure a homogeneous genetic background between all lines, all transgenes used, unless otherwise stated, were backcrossed to the *w^Dah^* stock for six generations. All experiments were carried out on mated females, unless otherwise stated.

### Use of mifepristone (RU486) to induce transgene expression by elavGS

For all experiments involving RU486 addition to fly food, the compound was dissolved in a stock solution of ethanol and added to the fly food while it was still liquid but had cooled to 50°C. The stock solution was added to the food, mixed well, dispensed into individual fly vials and allowed to cool to room temperature overnight before storage at 4°C. On the day of experiments, food vials were warmed to room temperature before being used. RU486 (Sigma, stock solution 100 mm in ethanol) was added to the food at a final concentration of 200 μm, with 2 ml/l ethanol used as the vehicle control condition. To induce gene expression with RU486, 24–48 h after eclosion, female flies carrying a heterozygous copy of elavGS and at least one UAS construct were transferred to vials containing either vehicle control food or food supplemented with 200-μm mifepristone (RU486). Flies were maintained on either control food or RU486-containing food throughout their lifespan.

### Lifespan analysis

Flies were raised at standard density in 200-ml bottles. After eclosion, flies were allowed to mate for 24–48 h. For each experiment at least 130–150 females per condition were split into groups of 15 and housed in vials containing SYA medium with or without RU486. Deaths were scored, and flies tipped onto fresh food three times a week. Data are presented as cumulative probability of survival, and survival rates were compared using log–rank tests and Cox proportional hazards. All lifespans were measured at 25°C, unless otherwise stated.

### Negative geotaxis assay

The climbing assay was performed as previously described (81). Briefly, 50 flies were housed in a glass-walled chamber 25-cm tall, and flies were tapped to the bottom and allowed to climb for 20 s before scoring. The numbers of flies in the top 5 cm, centre 15 cm, and bottom 5 cm were scored. A performance index was calculated for each time point and plotted using the following formula:

$$\kern5pc \mathrm{PI}=\frac{0.5\ast \left[\mathrm{Number}\ \left(\mathrm{top}\right)+\mathrm{Number}\ \left(\mathrm{middle}\right)+\mathrm{Number}\ \left(\mathrm{bottom}\right)+\mathrm{Number}\ \left(\mathrm{top}\right)-\mathrm{Number}\ \left(\mathrm{bottom}\right)\right]}{\mathrm{Number}\ \left(\mathrm{top}\right)+\mathrm{Number}\ \left(\mathrm{middle}\right)+\mathrm{Number}\ \left(\mathrm{bottom}\right)} $$

Statistical analysis was performed in R using ordinal logistics package.

### RT-qPCR

Total RNA was extracted from 20 to 25 fly heads per sample using TRIzol^®^ (GIBCO) according to the manufacturer’s instructions. The concentration of total RNA purified for each sample was measured using an Eppendorf BioPhotometer. One microgram of total RNA was then subjected to DNA digestion using DNase I (Ambion), immediately followed by reverse transcription using the SuperScript^®^ II system (Invitrogen) with oligo(dT) primers. Quantitative PCR was performed using the PRISM 7000 sequence–detection system (Applied Biosystems), SYBR^®^ Green (Molecular Probes), ROX Reference Dye (Invitrogen) and HotStarTaq (Qiagen) by following the manufacturer’s instructions. Each sample was analysed in duplicate, and the values are the mean of three independent biological repeats ± SEM. The primers used were as follows:

eIF1A ATCAGCTCCGAGGATGACGC + GCCGAGACAGACGTTCCAGA.

lap GCACTTGGACTATTTGGTGCAC + GCATAAATCGCTCATTGCCATATG.

### Western blotting

Fifteen flies per sample were flash frozen in liquid nitrogen. Heads were isolated by vortexing and separation through a small sieve. Protein samples were prepared by homogenizing heads in 2× SDS Laemmli sample buffer [4% SDS, 20% glycerol, 120 mm Tris-HCl (pH 6.8), 200 mm DTT with bromophenol blue] and boiled at 95°C for 10 min. Samples were separated on pre-cast 4–12% Invitrogen Bis-Tris gels (NP0322) or 10% Bis-Tris gels and blotted onto PVDF or nitrocellulose membrane (for VGlut) in Tris-glycine buffer supplemented with 20% methanol. Membranes were blocked in 5% milk in TBST (Tris-buffered saline with 0.1% Tween-20) for 1 h at room temperature (RT) and then incubated with primary antibodies overnight at 4°C. Primary antibody dilutions used were as follows: anti-actin, 1:10 000 (Abcam, ab1801); anti-VGlut, 1:10 000 (a gift from Dr A. DiAntonio, Washington University, St. Louis); and anti-PICALM, 1:1000 (Abcam ab127551). Secondary antibodies used were anti-rabbit and anti-mouse HRP (Abcam, ab6789 and ab6721) at 1:10 000 dilutions for 1 h at RT. Bands were visualized with Luminata Forte (Millipore) and imaged with ImageQuant LAS4000 (GE Healthcare Life Sciences). Quantification was carried out with ImageQuant software or ImageJ.

### Aβ_42_ ELISA

Five fly heads were homogenized in 50 μl GnHCl extraction buffer (5 M guanidinium HCl, 50 mm HEPES (pH 7.3), 1:10 dilution of protease inhibitor cocktail (Roche, P8340) and 5 mm EDTA) and centrifuged at 21 000 × *g* for 5 min at 4°C, and cleared supernatant retained as the total fly Aβ_42_ sample. Aβ_42_ levels were measured with an ELISA kit (Thermal Fisher, KHB3441), according to the manufacturer’s instructions, and total protein levels were measured with a Bradford assay (Bio-Rad protein assay reagent). The amount of Aβ_42_ in each sample was expressed relative to the total protein content (picograms per microgram of total protein). Data are expressed as the mean ± SEM obtained from three biological repeats for each genotype.

### Cross-linking of antibody to beads

50 μl of resuspended Dynabeads^®^ Protein G (Life Technologies, 10004D) were precipitated on a magnet for 1 min, the supernatant removed, and the beads washed 4 times in 1 ml of 0.2 M triethanolamine, pH 8.2. The beads were then resuspended in 1 ml of fresh 20 mm DMP (dimethyl pimelimidate dihydrochloride, Pierce #21666) in 0.2 M triethanolamine, pH 8.2 (5.4 mg DMP/ml buffer), together with the antibody, DVGlut (11 000) and PICALM (1:500), and incubated overnight at 4°C. The beads were then precipitated, and the cross-linking reaction stopped by addition of 1 ml of 50 mm Tris, pH 7.5 for 15 min. The beads were then washed with 1 ml PBS pH 7.4 and resuspended in PBS (pH 7.4) until required.

### Co-immunoprecipitation

Forty adult fly heads were isolated from *W^Dah^* flies and homogenized in 600-μl of lysis buffer [50 mm Tris (pH 7.5), 150 mm NaCl, 0.5% NP-40 (v/v), 0.1 mm MgCl_2_, 0.1 mm Na_3_VO_4_, 5 mm NEM, 1:10 dilution of protease inhibitor cocktail (Roche, P8340), 1:100 dilution of phosphatase inhibitor cocktail 2 (Sigma P5726) and 1 mm phenylmethylsulfonyl fluoride (PMSF)] and spun at 13 000 rpm (17 949 × g) for 15 min at 4°C. 50 μl of the supernatant was taken for ‘input fraction’, while 200 uL of the remaining supernatant was subjected to IP. The lysates were precleared with 10 uL of protein G dynabeads (Life Technologies, 10004D) for 10 min at 4°C and then incubated with 50 ul of the same beads cross-linked with anti-PICALM or anti-dVGlut antibody overnight at 4°C. A bead-only control was also run and treated like the IP samples. Beads were collected by a magnetic stand, and 200 μl of supernatant was removed (supernatant fraction). The beads were washed for five times with 1-ml PBS with 0.5% BSA (PBSB). The beads were then heated to 70°C for 5 min with 20 ul 2× SDS sample buffer and 200 mm DTT, and the supernatant was collected as the ‘IP fraction’ or ‘IgG fraction’ in the case of the bead-only control. Input and supernatant were diluted in 2× SDS sample buffer to a 1× final concentration with 200 mm DTT.

### Immunohistochemistry

Wandering third-instar larvae were dissected in HL3 solution and fixed in Bouin’s fixative (for VGlut and GluRII) or 4% paraformaldehyde (for Amph) for 20 min. Larvae were then rinsed in PBS with 0.2% Triton-100 (PBST) and blocked in 5% BSA in PBST for 1 h at RT and then incubated with primary antibodies overnight at 4°C and washed in PBST three times. Primary antibody dilutions used were as follows: anti-GluRIIA, 1:100 (8B4D2 obtained from the Developmental Studies Hybridoma Bank, University of Iowa, Iowa City, IA); anti-VGlut, 1:1000; anti-GluRIIB, 1:2500; anti-GluRIIC, 1:1000 (a gift from Dr A. DiAntonio, Washington University, St. Louis); and anti-Amph, 1:2500 (a gift from Dr A. Zelhof, Indiana University, Bloomington). Secondary antibodies were anti-HRP, 1:200 (Jackson ImmunoResearch, West Grove, PA); anti-rabbit Alexa Fluor 568, 1:1000 (Thermal Fisher, A11036); and anti-mouse Alexa Fluor 568, 1:1000 (Thermal Fisher, A11019), all incubated for 1 h at RT. After washing, larvae were mounted in Vectashield (Vector Laboratories, Burlingame, CA).

### Image acquisition and analysis

All images were acquired as stacks on a Zeiss LSM700 inverted confocal microscope using a 63× objective and are shown as maximum intensity projections of the complete *Z*-stack. The 10-μm stack was taken from muscles 7 and 6 of segments A2-A4. All images for one experiment were taken at the same microscope settings to reduce variability, and all larvae dissected were imaged. All images from one experiment were processed in the same way, setting the threshold so that all the background intensities would be the same across all samples. Mean fluorescence intensity of each slice of the NMJ was measured with ImageJ. Values shown are the averages for 5–10 NMJ ± SEM. Samples were compared by one-way ANOVA followed by Tukey’s *post hoc* test.

### Live imaging of glutamate release

Heterozygous D42-Gal4 > UAS-iGluSnFR L2 larvae were prepared for live imaging as described previously (50) and imaged on a Zeiss LSM880 airyscan confocal microscope. We immobilized larvae by gently squeezing them under a cover glass in halocarbon oil. Images were collected at a rate of 0.92 frame/s. A single plane was taken from the ventral nerve cord (VNC). All images for one experiment were taken at the same microscopy settings and motion-corrected using the Fiji plug-in, MoCo (82). The mean fluorescence intensity of a specific ROI per VNC was measured with Fiji for each time point.

Glutamate signals for each region of interest (ROI) were defined as deviations from the average fluorescence intensity inside each ROI in each frame, *Ft*, measured as a function of time (Δ*F/F* = (*F_t_* − *F*_0_)/*F*_0_), where F0 is minimal fluorescence intensity recorded. Glutamate ‘events’ were defined as Δ*F*/*F* > 0.1. Glutamate event onsets were set as the first frame in the rising phase of the signal. Glutamate event could be either single events or bursts. Glutamate ‘bursting’ was defined by a continuous glutamate transient with ≥2 glutamate events, with the offset of each burst set as the first local minimum in the trace when the falling F/F0 was within 0.2 of the rising onset. Glutamate bursting onsets for all glutamate events were set as the first frame in the rising phase of the signal. For glutamate bursts only the highest peak was scored.

Samples were compared by one-way ANOVA followed by Tukey’s *post hoc* test.

### Statistical analysis

Statistical analysis is described in each section above. The interaction between genotype and RU were analysed by Cox proportional hazards (for lifespans) and ordinal logistics analysis (for climbings) in R. One-way ANOVAs were carried using GraphPad Prism v8.0 software. Parameters are reported in the figure legends.

## Supplementary Material

movie1_ddaa125

movie2_ddaa125

movie3_ddaa125

movie4_ddaa125
